# Reliability of the nitrogen washin-washout technique to assess end-expiratory lung volume at variable PEEP and tidal volumes

**DOI:** 10.1186/2197-425X-2-10

**Published:** 2014-04-09

**Authors:** Jean-Christophe Richard, Céline Pouzot, Alfredo Morales Pinzón, Juan Sebastian Torres González, Maciej Orkisz, Bruno Neyran, Marcela Hernández Hoyos, Franck Lavenne, Claude Guerin

**Affiliations:** Service de Réanimation Médicale, Hôpital de la Croix Rousse, Hospices Civils de Lyon, 103 Grande Rue de la Croix Rousse, 69004 Lyon, France; CREATIS, INSERM 1044, CNRS 5220, Villeurbanne, France; Université LYON I, Université de Lyon, Lyon, France; Service SIAMU, VetAgro Sup, Campus Vétérinaire de Lyon, Marcy l′Etoile, France; EA 4174 Hémostase, Inflammation et Sepsis, Université de Lyon, VetAgro Sup, Marcy l′Etoile, France; Grupo Imagine, Grupo de Ingeniería Biomédica, Universidad de los Andes, Bogotá, Colombia; CERMEP, Imagerie du vivant, Hôpital Neurologique, Lyon, France

**Keywords:** ARDS, End-expiratory lung volume, Computed tomography, PEEP

## Abstract

**Background:**

End-expiratory lung volume measurement by the nitrogen washin-washout technique (EELV_WI-WO_) may help titrating positive end-expiratory pressure (PEEP) during acute respiratory distress syndrome (ARDS). Validation of this technique has been previously performed using computed tomography (EELV_CT_), but at mild PEEP levels, and relatively low fraction of inspired oxygen (FiO_2_), which may have insufficiently challenged the validity of this technique. The aims of this study were (1) to evaluate the reliability of EELV_WI-WO_ measurements at different PEEP and *V*_T_ during experimental ARDS and (2) to evaluate trending ability of EELV_WI-WO_ to detect EELV changes over time.

**Methods:**

ARDS was induced in 14 piglets by saline lavage. Optimal PEEP was selected during a decremental PEEP trial, based on best compliance, best EELV_WI-WO_, or a PEEP-FiO_2_ table. Eight *V*_T_ (4 to 20 mL · kg^-1^) were finally applied at optimal PEEP. EELV_WI-WO_ and EELV_CT_ were determined after ARDS onset, at variable PEEP and *V*_T_.

**Results:**

EELV_WI-WO_ underestimated EELV_CT_ with a non-constant linear bias, as it decreased with increasing EELV. Limits of agreement for bias were ±398 mL. Bias between methods was greater at high PEEP, and further increased when high PEEP was combined with low *V*_T_. Concordance rate of EELV changes between consecutive measurements was fair (79%). Diagnostic accuracy was good for detection of absolute EELV changes above 200 mL (AUC = 0.79).

**Conclusions:**

The reliability of the WI-WO technique is critically dependent on ventilatory settings, but sufficient to accurately detect EELV change greater than 200 mL.

**Electronic supplementary material:**

The online version of this article (doi:10.1186/2197-425X-2-10) contains supplementary material, which is available to authorized users.

## Background

Acute respiratory distress syndrome (ARDS) is characterized by a major decrease in lung aerated volume. End-expiratory lung volume measurement by the nitrogen washin-washout technique (EELV_WI-WO_) [1] is available at the bedside from an ICU ventilator, and may help titrating PEEP during mechanical ventilation of ARDS patients. Validation of this technique has been previously performed in mechanically ventilated patients using computed tomography (CT) as gold standard [2], but at relatively low PEEP levels (5 cm H_2_O), low fraction of inspired oxygen (FiO_2_) and respiratory rate (RR), and with tidal volume (*V*_T_) 8 ± 1 mL · kg^-1^ in the upper range of current experts’ recommendations for ARDS management [3]. Such ventilatory settings may have insufficiently challenged the validity of this technique, which requires a constant inhomogeneity in alveolar gas throughout the measurement, and may be less precise at FiO_2_ greater than 0.7 [1].

Furthermore, the WI-WO technique is particularly suitable for repeated EELV assessment, and hence to identify EELV trends, but has never been formally validated for this purpose.

The aims of this study were to evaluate (1) the reliability of EELV_WI-WO_ measurement at variable PEEP and *V*_T_, at high RR and FiO_2_ during experimental ARDS, using CT as a reference and (2) the trending ability of WI-WO technique to detect change in EELV associated with PEEP and *V*_T_ variations.

## Methods

This study was approved by our Institutional Review Board for the care of animal subjects (Comité d’experimentation animale de l′université Lyon I), and carried out in 14 pigs (28 ± 2 kg).

### Animal preparation

Pigs were anesthetized with propofol and fentanyl, tracheotomized and mechanically ventilated in volume-controlled mode, with constant inspiratory flow, *V*_T_ 10 mL · kg^-1^, inspired fraction of oxygen (FiO_2_) 0.21, zero end-expiratory pressure, and RR adjusted to achieve normocapnia using Engström Carestation^®^ ventilator (General Electric Healthcare, Madison, WI, USA). Muscle relaxation was obtained with pancuronium bromide. Right jugular vein was cannulated with a 3-lumen 8.5-Fr catheter for drug administration. Carotid artery was cannulated with an 8.5 Fr catheter. FiO_2_ was increased to 1 at the end of animal preparation.

### Measurements

Air flow was measured using a small volume pneumotachograph (PN 281637, Hamilton medical AG, Bonaduz, Switzerland). Pressure at the airway opening was measured using a connecting tube with lateral aperture connected between the endotracheal tube and the pneumotachograph. Signals of arterial blood pressure, pressure at the airway opening, and air flow were read by transducers (Becton Dickinson, Sandy, UT, USA), connected to an A/D card (MP 100; Biopac Systems, Santa Barbara, CA, USA), acquired at 200 Hz and analyzed with Acknowledge^®^ software (Biopac Systems, Santa Barbara, CA, USA). Tracheal pressure was measured through an air filled catheter introduced down the endotracheal tube, positioned 2 cm distal to the tube tip, and connected to the ventilator, to obtain alveolar pressure [4].

EELV_WI-WO_ was assessed by the ventilator, by using the nitrogen washout/washin technique [1], from continuous measurement of end-tidal O_2_ and CO_2_ during a 0.1 change of FiO_2_ using pediatric sensors (Pedi-lite+, Dahtex-Ohmeda Inc, Madison, WI, USA). The average value of the washout and washin measurements during 1 to 0.9 and 0.9 to 1 FiO_2_ changes was given by the ventilator.

EELV_CT_ was calculated using lung CT, as previously described [2]. CT calibration using the manufacturer phantom was performed before each CT study. The CT scanner (Biograph mCT/S, Siemens, Munich, Germany) was set as follows: interval 1 mm, voltage 120 kV, pitch 1.2 mm, and field of view 300 mm. Whole lung CT images were taken during 15 s end-expiratory. CT raw data were reconstructed as 1-mm-thick contiguous slices using a medium smooth filter (B31f). Image segmentation was manually performed over the whole lung using Turtleseg^®^ software [5, 6] (http://www.turtleseg.org). Gas volume in each lung voxel was computed from the CT number using the following formulas [2]:

Gas volume = 0 for lung voxels with CT number > 0.

EELV_CT_ was computed as the sum of gas volume in all the voxels defined by lung segmentation.

Expected EELV on zero end-expiratory pressure was deemed as 33 mL · kg^-1^ body weight as previously published in normal anesthetized pigs [7].

### Protocol

ARDS was performed by saline lavage at ventilatory settings mentioned above. Intra-tracheal instillations of 1,000 mL aliquots of 0.9% sodium chloride warmed at 37°C were repeated until PaO_2_/FiO_2_ ratio was <100 mmHg. RR may be increased up to 35 breaths per min to maintain pH above 7.20, then kept constant except at the end of experiment, where at the highest *V*_T,_ it was decreased to maintain peak airway pressure below 100 cm H_2_O.

Then, PEEP was set to 20 cm H_2_O, *V*_T_ to 6 mL · kg^-1^, and a recruitment maneuver was performed by applying a continuous airway pressure of 40 cm of H_2_O over 40 s. A decremental PEEP trial was then performed from 20 to 2 cm H_2_O by 2 cm H_2_O steps of 10 min each. At the end of the decremental PEEP trial, animals were randomized into three PEEP groups, for which PEEP level was set according to either highest compliance (*n* = 4), or highest EELV_WI-WO_ (*n* = 5), or PEEP-FiO_2_ table (*n* = 4) [8]. This randomization was used to deliver a wide PEEP range during the final part of the study, in order to obtain multiple combinations of PEEP and *V*_T_, so as to perform a multivariate analysis adjusted for PEEP and *V*_T_. One pig died just after the PEEP trial before randomization, and was kept in the final analysis. The selected PEEP was applied for 1 h, and *V*_T_ was adjusted to maintain plateau pressure of the respiratory system ≤30 cm of H_2_O.

After 1 h of applied selected PEEP, eight levels of *V*_T_ (4, 5, 6, 7, 8, 10, 15, 20 mL · kg^-1^) ranging from 100 to 625 mL, were applied for 2 min leaving PEEP level unchanged. EELV_WI-WO_ and EELV_CT_ were measured immediately after ARDS onset, at the end of each PEEP step during the PEEP trial, and at the end of each *V*_T_ change. A 15-s end-inspiratory pause was performed to check the absence of air leak in each experimental condition. Some experimental conditions were not available since pneumothorax occurred in several pigs at high *V*_T_ or since EELV_WI-WO_ values were lacking for technical reasons, ending up in 218 data points in final analysis (see Additional file 1: Table S1 for description of lacking data points).

### Statistical analysis

Statistical analyses were performed using R software [9], with packages nlme [10], MethComp [11], pROC [12], OptimalCutpoints [13], and multcomp [14]. Values were expressed as mean ± standard deviation (SD). The level of statistical significance was set below 0.05.

EELV_WI-WO_ and EELV_CT_ were compared using a linear mixed-effects model, and Bland and Altman representation [15]. Limits of agreement were computed using alternating regression [16] since bias was non-constant and the experimental design involved repeated measurements.

To control for an effect of confounding variables on the bias, a linear mixed-effects model was built using PEEP, *V*_T_, EELV_CT_ at ARDS onset and their interactions as factors with fixed effect, pigs as factor with random effect [17], and bias as dependent variable. Model simplification was performed using a backward stepwise algorithm.

Percentage error was computed as × SD_Bias_/mean_EELV_[18]. As percentage error was not reported in the two previously published studies that compared EELV_WI-WO_ and EELV_CT_[2, 19], Cartesian data of these studies were reanalyzed, being uplifted using a scientific program allowing extraction of individual data points from a digitalized graph (DataThief III^®^[20]) as follows: a digital copy of each regression plot was analyzed with DataThief from the portable document format file of the journal articles, and the extracted data were exported as two columns of *X*-*Y* coordinates, with each row representing an extracted data point, allowing computation of percentage error of each study.

Changes in EELV between consecutive measurements were computed for EELV_WI-WO_ (ΔEELV_WI-WO_) and EELV_CT_ (ΔEELV_CT_). Ability of the WI-WO technique to track changes in EELV was assessed using four-quadrant and polar plots. The four-quadrant plot relates ΔEELV_WI-WO_ and ΔEELV_CT_, with upper right and lower left quadrants being quadrants of agreement (in which both EELV_WI-WO_ and EELV_CT_ have the same directional changes) and lower right and upper left quadrants being quadrants of disagreement (in which EELV_WI-WO_ and EELV_CT_ have opposite directional changes). Concordance rate was defined as the percentage of data points falling into one of the two quadrants of agreement, expressed as a percentage of the total number of data points [21]. The main drawback of the four-quadrant plot is the lack of quantification of the distance between each data point and the line of identity, leading to the development of polar plot analysis [21]. Polar plot is obtained by a 45° clockwise rotation of the four-quadrant plot, changing the dimensions of the radius to mean ΔEELV [22], lining up the line of identity along the horizontal axis. Data points with positive and negative directional changes are located on the right and the left side of the polar plot, respectively, and the polar angle represents the angle of each data point with line of identity. A 0° polar angle depicts a perfect agreement between ΔEELV_WI-WO_ and ΔEELV_CT_, while polar angles in the range 45° to 135° and 225° to 315° depict disagreement between directional changes of EELV_WI-WO_ and EELV_CT_. The following variables are computed from polar plots: (1) angular bias as the mean angle between all data points and polar axis [21], reflects the difference in calibration between the reference and test methods; (2) radial limits of agreement as the radial sector containing 95% of the data points, after conversion of negative deflections to positive ones, is a polar version of the 95% confidence limits and is similar to the limits of agreement in Bland and Altman analysis [21].

Bias and angular bias were compared to zero using Mann-Whitney *U* test. Multiple comparisons were performed with Dunnett’s test using PEEP 0 as a reference.

The ability of the WI-WO technique to detect a change in EELV greater than 100, 150, 200, 250, and 300 mL was tested by computations of area under receiver operating characteristic (AUC) curve. The optimal cut-off points were computed using the Youden J statistic.

## Results

**Figure 1:**
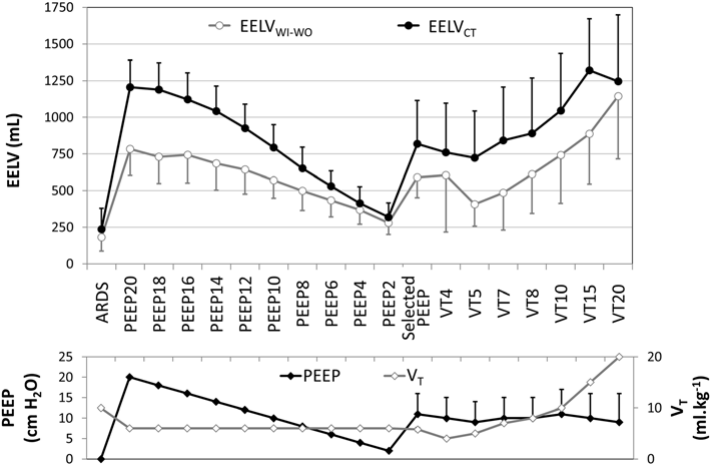
**EELV**_**WI-WO**_ **and EELV**_**CT**_ **at each experimental condition (upper panel), with corresponding RR, PEEP, and** ***V***_**T**_ **(lower panel).** Values are mean ± standard deviation. ARDS, experimental acute respiratory distress syndrome; EELV_WI-WO_, end-expiratory lung volume assessed with the nitrogen washin-washout technique; EELV_CT_, end-expiratory lung volume assessed by computed tomography; optimal PEEP, optimal PEEP level according to one of the three methods (see text for details); PEEP, positive end-expiratory pressure; RR, respiratory rate; *V*_T_, tidal volume.

Ventilatory settings and arterial blood gases during the whole experiment are reported in Additional file 2: Table S2. Figure 1 depicts the evolution of EELV_WI-WO_ and EELV_CT_ over time. Immediately after ARDS onset, EELV_CT_ was very low (236 ± 143 mL (25% ± 15% of its theoretical value, range 104 to 668 mL)), and increased to 1,206 ± 185 mL (range 957 to 1,528 mL) at PEEP 20.

EELC_CT_ and EELV_WI-WO_ values were very close at PEEP 0, but their difference progressively increased with the PEEP level.

### Comparison of EELV_WI-WO_ and EELV_CT_

**Figure 2:**
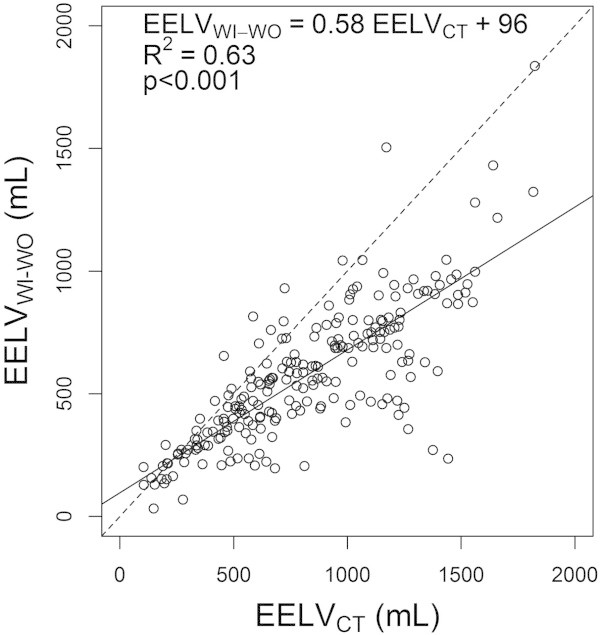
**Relationship between EELV**_**WI-WO**_ **and EELV**_**CT**_ **.** Each symbol represents a concomitant measurement of end-expiratory lung volume assessed with either nitrogen washin-washout technique (EELV_WI-WO_) or computed tomography (EELV_CT_). Solid line is the regression line. Dashed line is the line of identity.

**Figure 3:**
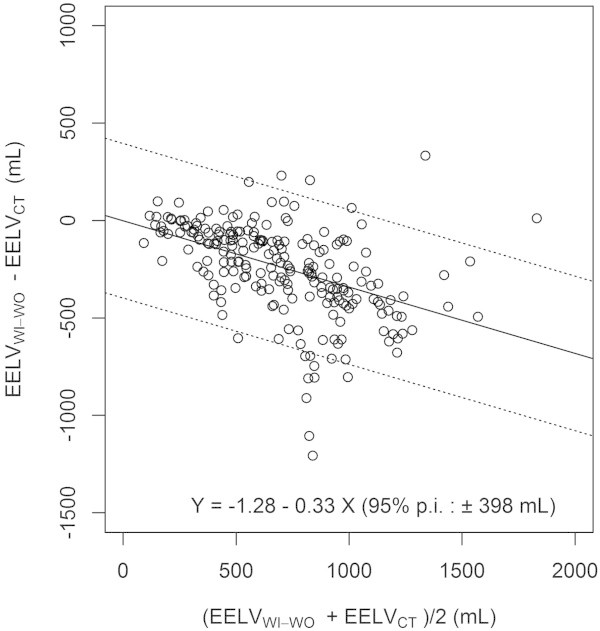
**Bias and limits of agreement between EELV**_**CT**_ **and EELV**_**WI-WO**_ **, using Bland and Altman representation.** Each symbol represents a concomitant measurement of EELV_WI-WO_ and EELV_CT_. Horizontal continuous line and horizontal broken lines are the mean bias and 95% prediction interval limits of the bias between EELV_WI-WO_ and EELV_CT,_ respectively. EELV_WI-WO_, end-expiratory lung volume assessed with the nitrogen washin-washout technique; EELV_CT_, end-expiratory lung volume assessed by computed tomography; 95% p.i., 95% prediction interval of the bias between EELV_WI-WO_ and EELV_CT_.

EELV_WI-WO_ and EELV_CT_ were significantly correlated (*R*^2^ = 0.63, *p* < 0.001). The regression equation between EELV_WI-WO_ and EELV_CT_, had an intercept of 96 mL (*p* < 0.001) and a slope of 0.58 mL^-1^ (*p* < 0.001; Figure 2). Bland and Altman representation exhibited a non-constant bias, decreasing toward more negative values as mean EELV increased (Figure 3). The difference between EELV_WI-WO_ and EELV_CT_ was related to their mean value by the following equation:

1

**Figure 4:**
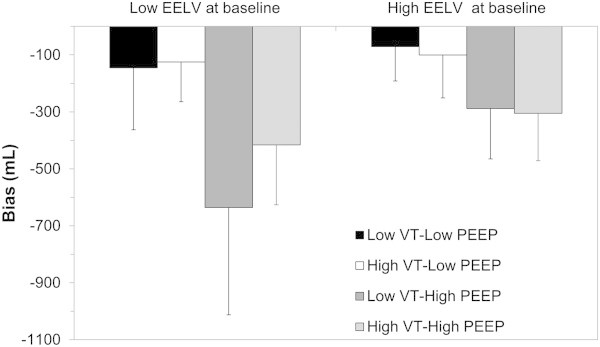
**Interaction plot between** ***V***_**T**_ **, PEEP, and EELV**_**Base**_ **.** *V*_T_, PEEP, and EELV_Base_ were classified as high or low, as a function of their relationship with their median value. The following cut-off values were identified: *V*_T_ 170 mL; PEEP 10 cm H_2_O; EELV_Base_ = 157 mL. Bars are mean values, and error bars, standard deviations. Bias, mean difference between EELV_WI-WO_ and EELV_CT_ in each subgroup; EELV_Base_, end-expiratory lung volume at baseline (ARDS onset, PEEP 0); EELV_WI-WO_, end-expiratory lung volume assessed with the nitrogen washin-washout technique; EELV_CT_, end-expiratory lung volume assessed by computed tomography; PEEP, positive end-expiratory pressure; *V*_T_, tidal volume.

**Figure 5:**
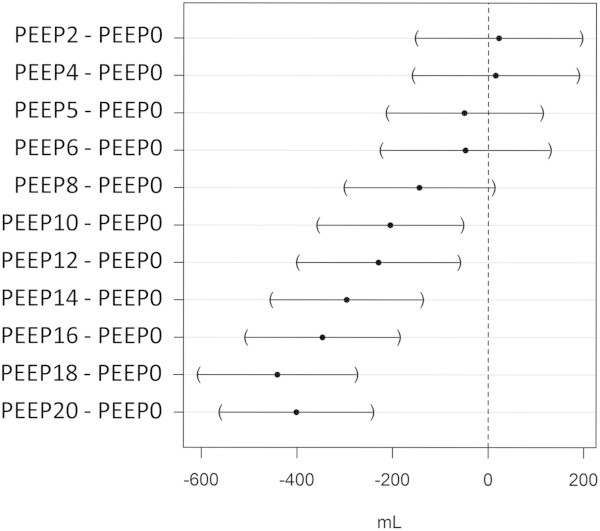
**Mean difference in bias between each PEEP level from 2 to 20 and PEEP 0 cm H**_**2**_ **O.** Closed circles are mean differences and bars are 95% confidence intervals.

Limits of agreement of the bias were ±398 mL (Figure 3), and percentage error was computed to 57%. A significant interaction between PEEP, *V*_T_, EELV baseline value on the bias between methods was identified (Table 1) and reported (Figure 4). The bias between methods was strongly influenced by PEEP level, increasing at higher PEEP regardless the *V*_T_ level. The bias further increased when high PEEP was combined to low *V*_T_, when EELV at baseline was low.The bias at PEEP 0 amounted to -54 ± 101 mL, was not significantly different from 0, and was compared to bias values at higher PEEP. As shown in Figure 5, the bias at PEEP 10 and higher did significantly differ from PEEP 0, while non-significant differences were found for lower PEEP.

**Table 1:** **Statistical modeling of the bias between EELV**_**WI-WO**_ **and EELV**_**CT**_ **as a function of confounding variables**

	AIC	Statistical significance
Model 1: No explanatory variable	2992	
Model 2: Adjusting for *V*_T_	2994	*V*_T_: *p* = 0.55
Model 3: Adjusting for PEEP	2868	PEEP: *p* < 0.0001
Model 4: Adjusting for EELV_Base_	2980	EELV_Base_: *p* < 0.0001
Model 5: Final model adjusting for *V*_T_, PEEP, EELV_Base_ and their three-way interaction	2821	three-way interaction: *p* < 0.0001
		*V*_T_ × PEEP interaction: *p* < 0.0001
		*V*_T_ × EELV_Base_: *p* < 0.001
		PEEP × EELV_Base_: *p* < 0.0001
		*V*_T_: *p* < 0.001
		PEEP: *p* < 0.0001
		EELV_Base_: *p* < 0.05

*AIC,* Akaike information criterion; *EELV*, end-expiratory lung volume; *EELV*_*Base*,_ EELV baseline value (ARDS onset, PEEP 0 cm H_2_O); PEEP, positive end-expiratory pressure; *V*_*T*,_ tidal volume.

### Assessment of trending ability of the WI-WO technique

**Figure 6:**
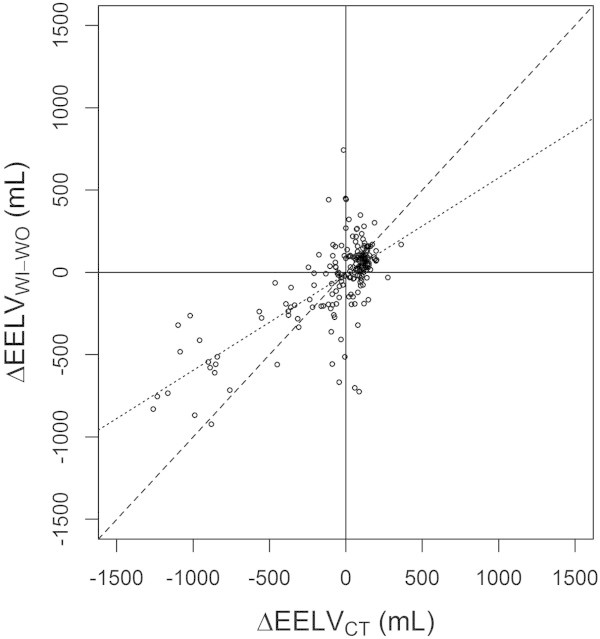
**Four quadrants plot relating ΔEELV**_**WI-WO**_ **with ΔEELV**_**CT**_ **between consecutive measurements.** Continuous black lines are quadrant limits. Dotted line is the regression line. Dashed line is the line of identity. Each data point is the change in end-expiratory lung volume (EELV) between consecutive measurements assessed with the nitrogen washin-washout technique (ΔEELV_WI-WO_) or computed tomography (ΔEELV_CT_).

ΔEELV_WI-WO_ values adequately tracked ΔEELV_CT_ changes against time (Figure 6). Concordance rate over all measurements amounted to 79%, and slightly increased after exclusion of small changes in EELV which do not reflect trending ability (Table 2).

**Table 2:** **Concordance rate, angular bias, and radial limits of agreement in different data subsets**

	No exclusion zone	Exclusion threshold 100 mL	Exclusion threshold 150 mL	Exclusion threshold 200 mL	Exclusion threshold 300 mL
Concordance rate	79%	82%	82%	86%	86%
Angular bias ± SD (°)	-4 ± 37	3 ± 25	6 ± 25	1 ± 26	-1 ± 25
Radial limits of agreement (°)	±78	±51	±48	±48	±50

Exclusion threshold refers to exclusion of data points with change in end-expiratory lung volume between consecutive time points below or equal to 100, 150, 200, and 300 mL, respectively. *SD*, standard deviation.

**Figure 7:**
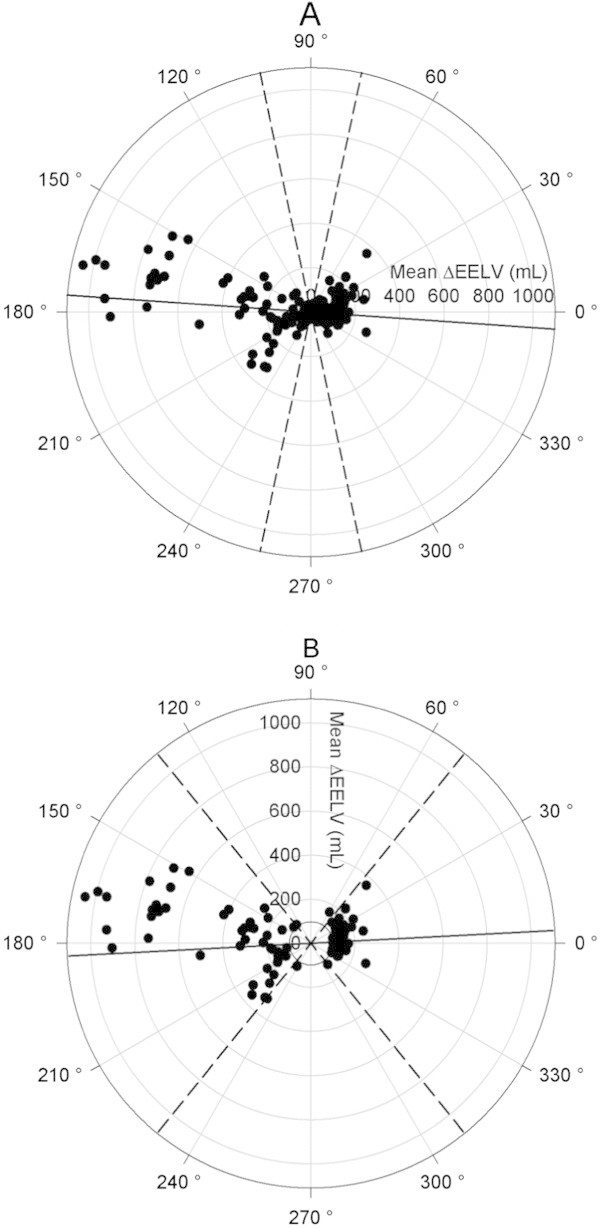
**Polar plots assessing trending ability of EELV**_**WI-WO**_ **to track changes in EELV.** Panel A refers to the whole set of measurements, and panel B is restricted to data related to changes in EELV greater than 100 mL since a small change in EELV does not reflect trending ability but mainly random error measurement. The radial axis joining 0 to 180° is a 45° clockwise rotation of the line of identity in the four-quadrant plots, and represents agreement. The better the agreement between ΔEELV measurements, the closer data pairs will lie along the horizontal radial axis. The distance from the center of each plot represents the mean change in EELV between methods (mean ΔEELV) at each consecutive time point. Data points located between 315° and 45° refer to time points in which both EELV_CT_ and EELV_WI-WO_ increased (upper right quadrant of the four quadrant plot), while data points located between 135° and 225° refer to consecutive time points in which both EELV_CT_ and EELV_WI-WO_ decreased (lower left quadrant of the four quadrant plot). Data points located between 45 and 135° or 225 and 315° correspond to disagreement in the directional change of EELV between the washin-washout technique and computed tomography. Continuous line represents the angular bias, while dashed lines represent radial limits of agreement. EELV_WI-WO_, end-expiratory lung volume assessed with the nitrogen washin-washout technique; EELV_CT_, end-expiratory lung volume assessed by computed tomography; ΔEELV, change in EELV between consecutive measurements.

Results of the polar plot analysis are reported in Figure 7, and in Table 2. The angular bias amounted to -4° ± 37° for all measurements. After exclusion of EELV changes ≤100 mL, the angular bias amounted to 3° ± 25°, and was not statistically different from 0. Radial limits of agreement were wide when all measurements were taken into account (±78°), but where narrowed to ±51° after exclusion of EELV changes ≤100 mL. Increasing the exclusion threshold of EELV changes up to 300 mL did not improve the radial limits of agreement (Table 2). Diagnostic performance of EELV_WI-WO_ to detect absolute EELV changes greater than 100, 150, 200, 250, and 300 mL is presented in Table 3. Diagnosis accuracy was fair for detection of absolute EELV changes above 200 mL (AUC 0.79 (CI 95% 0.70 to 0.89)), and good for detection of absolute EELV changes above 300 mL (AUC 0.89 (CI 95% 0.83 to 0.95)).

**Table 3:** **Diagnostic performance of EELV**_**WI-WO**_ **to detect variations in ELLV**_**CT**_ **at different thresholds**

ΔEELV_CT_ threshold for AUC computation (mL)	AUC (CI 95%)	Optimal ΔEELV_WI-WO_ cut-off (mL)	Se	Sp	PPV	NPV	PLR	NLR	Youden index
100	0.58 (0.50 to 0.66)	42	0.84	0.33	0.57	0.66	1.25	0.48	0.17
150	0.73 (0.64 to 0.81)	166	0.65	0.75	0.46	0.87	2.61	0.47	0.40
200	0.79 (0.70 to 0.89)	166	0.80	0.75	0.42	0.94	3.26	0.27	0.55
250	0.87 (0.79 to 0.94)	169	0.90	0.77	0.41	0.97	3.89	0.13	0.67
300	0.89 (0.83 to 0.95)	169	0.93	0.77	0.41	0.98	4.05	0.09	0.70

*AUC*, area under receiver operating characteristic curve; CI 95%, 95% confidence interval; *ΔEELV*_*CT*_, absolute change in end-expiratory lung volume assessed by computed tomography between consecutive time points; *ΔEELV*_*WI-WO*_ *,* absolute change in end-expiratory lung volume assessed with the nitrogen washin-washout technique between consecutive time points; *NLR*, negative likehood ratio; *NPV*, negative predictive value; *PLR*, positive likehood ratio; *PPV*, positive predictive value; *Se*, sensitivity, *Sp*, specificity.

## Discussion

The main findings of the present study are that, in experimental ARDS, (1) EELV_WI-WO_ underestimates EELV_CT_, and this underestimation increases linearly as EELV increases; (2) this underestimation is dependent on ventilatory settings (mainly PEEP); (3) the precision of this technique is poor with a percentage error as high as 57%; (4) this technique is however reliable to detect an EELV change greater than 200 mL.

A formal comparison between EELV_WI-WO_ EELV_CT_ has already been performed in a pig model of pleural effusion [19] and in mechanically ventilated patients [2]. While Chiumello et al. found a slightly positive constant bias between EELV_WI-WO_ and EELV_CT_[2], and Graf et al. a slightly negative constant bias, a non-constant linear bias was found in the present study. Beside differences in species, experimental protocol or mechanism of lung injury, the likely explanation of this discrepancy is related to the higher PEEP level applied in the present study (up to 20 cm H_2_O), while PEEP was set to 5 cm H_2_O in the human study [2] and ≤10 cm H_2_O in the animal study [19]. The dependence of bias on PEEP level was further emphasized in our study by multivariate analysis (Table 1, Figure 4), more specifically for PEEP levels greater than 10 cm H_2_O. Finally, a detrimental effect of PEEP levels greater than 16 cm H_2_O on EELV_WI-WO_ accuracy was also recently pointed out by Dellamonica et al. [23]. Nevertheless, in spite of a non-constant bias, the true EELV value may still be assessed by the WI-WO technique using Equation 1, as pointed out by Bland and Altman [24].

The detrimental effect of high PEEP levels on the bias may have multiple explanations. First, the WI-WO technique measures volume of lung regions that may be reached by nitrogen (and hence are ventilated), while CT measures aeration of both ventilated and non-ventilated regions. One could then hypothesize that regional ventilation (and hence nitrogen during EELV measurement) at high PEEP may be preferentially directed toward non-overinflated regions and that overinflated regions may not be detected in the EELV measurement. However, this explanation is not supported by our data, since the amount of overinflated area was very low even at the highest PEEP level in our study (1.1 ± 0.89 mL). Nevertheless, the observed difference in EELV between methods at high PEEP may be related to the fact that the WI-WO method measures a functional EELV, while CT measures anatomical EELV. Occurrence of leaks at high PEEP is another hypothesis to explain the non-constant bias, but this explanation is unlikely since plateau pressure was maintained during the prolonged end-inspiratory pauses performed for CT acquisition. Finally, there may be insufficient nitrogen mixing within the aerated lung, during the time allocated for measurement by the ventilator, since animals were ventilated at high RR and low *V*_T_, which may have increased the time to reach equilibrium during the WI-WO measurement. An extended time between experimental stages and/or a lengthened WI-WO period may have narrowed the bias between methods.

Regarding precision, comparison between aforementioned studies, using limits of agreement is hindered by heterogeneity of EELV values across studies. Assessment of percentage error may overcome this problem, but was unavailable in the two previously published studies [2, 19]. Using Data Thief 3.0 to extract Cartesian data from these studies, we have computed from the author’s published figures, the percentage error which amounted to 28% in the human study and 46% in the pig study, versus 57% in our study. The relatively lower precision computed from our data may be a consequence of ventilatory settings, that may have particularly challenged the validity of the WI-WO technique. Indeed, FiO_2_ greater than 0.7 precludes the computation of the respiratory quotient (RQ) required for EELV measurements, and a default RQ of 0.85 is assumed by the Engström Carestation^®^ ventilator.

However, using a metabolically active lung model, Olegard et al. have nicely demonstrated that errors in RQ computation have a negligible effect on the precision of EELV measurements [1], suggesting that the high FiO_2_ used in our study may have only marginally influenced our results. Regarding trending, the lack of significant angular bias suggests that calibration of EELV_WI-WO_ is in agreement with the reference method. However, the relatively wide radial limits of agreement suggest that external factors may account for the variability of the relationship between ΔEELV_WI-WO_ and ΔEELV_CT_. We could speculate that this phenomenon is mainly related to the effect of PEEP on the bias between EELV_WI-WO_ and ΔEELV_CT_.

Our study has several strengths. Since multiple combinations of PEEP and *V*_T_ were evaluated, a systematic analysis of the effect of ventilator parameters on the reliability of the technique could be performed using multivariate analysis, and was able to identify the PEEP level as an independent risk factor for measurement error. Furthermore, this is the first study having assessed trending ability of the WI-WO technique, and provided with cut-off values above which EELV changes may be considered as meaningful. Finally, the present study, while performed with particularly challenging ventilatory settings (high FiO_2_, high PEEP, high RR, and low *V*_T_), demonstrates that the validity of the WI-WO technique may be extended to the sickest ARDS patients.

Our study has nevertheless some methodological issues that must be addressed. First, it was conducted using pediatric sensors for EELV_WI-WO_ measurements, which may be less accurate for the highest *V*_T_. Nevertheless, a subset analysis after exclusion of *V*_T_ > 300 mL led to similar results (Additional file 3: Figure S1). Furthermore, the bias was not increased at high *V*_T_, as compared to low *V*_T_, as shown by our interaction plot (Figure 4). Another limitation, in the perspective of extrapolation of these results to ARDS patients, is related to the relatively low EELV achieved in the pigs of this study in some experimental conditions. However, 62% of the EELV_CT_ measurements were greater than the first EELV quartile observed at low PEEP in a recent study on 30 ARDS patients [23], suggesting that most of the measurements performed in our study are in the range of clinically plausible values for EELV in ARDS patients. Another potential limitation is that the ARDS model used in the present study is particularly recruitable with PEEP. However, 50% of ARDS patients are considered recruiters by PEEP [23, 25], and early ARDS share similar features as saline lavage regarding response to PEEP. The 2-min interval between *V*_T_ changes and EELV measurements may have been too short for CO_2_ equilibration and achievement of both progressive recruitment and blood flow redistribution. However, a subset analysis limited to data acquired during the PEEP trial (with 10 min between measurements) led to similar results (see Additional file 4: Figure S2).

The present study may have important clinical implication. Indeed, as shown in Table 3, a change in EELV_WI-WO_ greater than 166 mL would give an important clue to the clinician that the true EELV has changed by more than 200 mL. Furthermore, despite the non-constant bias of the EELV measurement by the WI-WO technique, the true EELV value may still be assessed using Equation 1, provided that the absolute EELV value is relevant for the clinician [24].

## Conclusion

The reliability of the WI-WO technique is critically dependent on ventilatory settings, but sufficient to accurately detect EELV change over time greater than 200 mL.

## Electronic supplementary material

Additional file 1: Table S1: Reasons for lack of data as a function of each of the 3 experimental stages. Values are number of lacking data/total number of data (%). EELV_WI-WO_, end-expiratory lung volume assessed with the nitrogen washout-washin technique; EELV_CT_, end-expiratory lung volume assessed by computed tomography; *V*_T_, tidal volume. (DOCX 15 KB)

Additional file 2: Table S2: Ventilatory settings and arterial blood gases in each experimental condition. Values are number of mean ± standard deviation (range). ALI, acute lung injury onset; PEEP, positive end-expiratory pressure; RR, respiratory rate; *V*_T_, tidal volume. (DOCX 17 KB)

Additional file 3: Figure S1: Bias and limits of agreement between EELV_CT_ and EELV_WI-WO_, using Bland and Altman representation in a subset of the data (exclusion of *V*_T_ > 300 mL). Each symbol represents a concomitant measurement of EELV_WI-WO_ and EELV_CT_. Horizontal continuous line and horizontal broken lines are the mean bias and 95% prediction interval limits of the bias between EELV_WI-WO_ and EELV_CT,_ respectively. EELV_WI-WO_, end-expiratory lung volume assessed with the nitrogen washout-washin technique; EELV_CT_, end-expiratory lung volume assessed by computed tomography; 95% p.i., 95% prediction interval of the bias between EELV_WI-WO_ and EELV_CT_; *V*_T_, tidal volume. (DOCX 278 KB)

Additional file 4: Figure S2: Bias and limits of agreement between EELV_CT_ and EELV_WI-WO_, using Bland and Altman representation in a subset of the data, acquired during the PEEP trial (with 10 min between measurements). Each symbol represents a concomitant measurement of EELV_WI-WO_ and EELV_CT_. Horizontal continuous line and horizontal broken lines are the mean bias and 95% prediction interval limits of the bias between EELV_WI-WO_ and EELV_CT,_ respectively. EELV_WI-WO_, end-expiratory lung volume assessed with the nitrogen washin-washout technique; EELV_CT_, end-expiratory lung volume assessed by computed tomography; 95% p.i., 95% prediction interval of the bias between EELV_WI-WO_ and EELV_CT_; *V*_T_, tidal volume. (DOCX 137 KB)

## Abbreviations

ARDS: acute respiratory distress syndrome
AUC: area under curve
ΔEELV_WI-WO_: change in end-expiratory lung volume between consecutive measurements by the nitrogen washin-washout technique
ΔEELV_CT_: change in end-expiratory lung volume between consecutive measurements by computed tomography
EELV: end-expiratory lung volume
EELV_WI-WO_: end-expiratory lung volume measurement by the nitrogen washin-washout technique
EELV_CT_: end-expiratory lung volume measurement by computed tomography
FiO_2_: fraction of inspired oxygen
PEEP: positive end-expiratory pressure
RR: respiratory rate
SD: standard deviation
*V*_T_: tidal volume
WI-WO: nitrogen washin-washout
