# Supplementary material for: Reliability of the nitrogen washin-washout technique to assess end-expiratory lung volume at variable PEEP and tidal volumes
Source: Intensive Care Med Exp. 2014 Apr 9;2:10. doi: 10.1186/2197-425X-2-10 (PMC4512977; doi:10.1186/2197-425X-2-10)
Supplement: Supplementary file 1 — Additional file 1: Table S1: Reasons for lack of data as a function of each of the 3 experimental stages. Values are number of lacking data/total number of data (%). EELVWI-WO, end-expiratory lung volume assessed with the nitrogen washout-washin technique; EELVCT, end-expiratory lung volume assessed by computed tomography; V T, tidal volume. (DOCX 15 KB) [file 40635_2013_12_MOESM1_ESM.docx]

**Additional file 1**

**File name**: Additional file 1

**File format**: .pdf

| Reasons for lack of data | ARDS onset | PEEP trial | Variable V_T_ trial |
| --- | --- | --- | --- |
| EELV_WI-WO_ unavailable | 0/14 (0%) | 8/140 (6%) | 21/112 (19%) |
| EELV_CT_ unavailable | 0/14 (0%) | 0/140 (0%) | 6/112 (5%) |
| Pneumothorax occurrence | 0/14 (0%) | 0/140 (0%) | 13/112 (12%) |

**Title of data**: Reasons for lack of data as a function of each of the 3 experimental stages.

**Description of data**: Values are number of lacking data/total number of data (%)

EELV_WI-WO_ = end-expiratory lung volume assessed with the nitrogen washout-washin technique; EELV_CT_ = end-expiratory lung volume assessed by computed tomography; V_T_.= tidal volume.
