# Supplementary material for: Reliability of the nitrogen washin-washout technique to assess end-expiratory lung volume at variable PEEP and tidal volumes
Source: Intensive Care Med Exp. 2014 Apr 9;2:10. doi: 10.1186/2197-425X-2-10 (PMC4512977; doi:10.1186/2197-425X-2-10)
Supplement: Supplementary file 2 — Additional file 2: Table S2: Ventilatory settings and arterial blood gases in each experimental condition. Values are number of mean ± standard deviation (range). ALI, acute lung injury onset; PEEP, positive end-expiratory pressure; RR, respiratory rate; V T, tidal volume. (DOCX 17 KB) [file 40635_2013_12_MOESM2_ESM.docx]

**Additional file 2**

**File name**: Additional file 2

**File format**: .pdf

|  | ALI | PEEP  20 | PEEP  18 | PEEP  16 | PEEP  14 | PEEP  12 | PEEP  10 | PEEP  8 | PEEP  6 | PEEP  4 | PEEP  2 | Selected PEEP | V_T_4 | V_T_5 | V_T_7 | V_T_8 | V_T_10 | V_T_15 | V_T_20 |
| --- | --- | --- | --- | --- | --- | --- | --- | --- | --- | --- | --- | --- | --- | --- | --- | --- | --- | --- | --- |
|  |  |  |  |  |  |  |  |  |  |  |  |  |  |  |  |  |  |  |  |
| V_T_ (ml.kg^-1^) | 10 ± 0  (10-10) | 6 ± 0  (6-6) | 6 ± 0  (6-6) | 6 ± 0  (6-6) | 6 ± 0  (6-6) | 6 ± 0  (6-6) | 6 ± 0  (6-6) | 6 ± 0  (6-6) | 6 ± 0  (6-6) | 6 ± 0  (6-6) | 6 ± 0  (6-6) | 6 ± 0  (5-6) | 4 ± 0  (4-4) | 5 ± 0  (5-5) | 7 ± 0  (7-7) | 8 ± 0  (8-8) | 10 ± 0  (10-10) | 15 ± 0  (15-15) | 20 ± 0  (20-20) |
| RR (min^-1^) | 22 ± 7  (15-35) | 35 ± 0  (35-35) | 35 ± 0  (35-35) | 35 ± 0  (35-35) | 34 ± 3  (22-35) | 34 ± 3  (22-35) | 34 ± 3  (22-35) | 34 ± 4  (22-35) | 34 ± 4  (22-35) | 35 ± 0  (35-35) | 35 ± 0  (35-35) | 34 ± 4  (22-35) | 34 ± 2  (30-35) | 34 ± 2  (30-35) | 34 ± 2  (30-35) | 34 ± 2  (30-35) | 34 ± 2  (30-35) | 34 ± 2  (30-35) | 25 ± 8  (15-35) |
| PEEP (cm H_2_O) | 0 ± 0  (0-0) | 20 ± 0  (20-20) | 18 ± 0  (18-18) | 16 ± 0  (16-16) | 14 ± 0  (14-14) | 12 ± 0  (12-12) | 10 ± 0  (10-10) | 8 ± 0  (8-8) | 6 ± 0  (6-6) | 4 ± 0  (4-4) | 2 ± 0  (2-2) | 11 ± 5  (5-20) | 10 ± 5  (5-20) | 9 ± 5  (5-20) | 10 ± 5  (5-20) | 10 ± 5  (5-20) | 11 ± 6  (5-20) | 10 ± 6  (5-20) | 9 ± 7  (5-20) |
| pH | 7.24±0.06  (7.14-7.33) |  |  |  |  |  |  |  |  |  |  | 7.27±0.14  (6.95-7.42) |  |  |  |  |  |  |  |
| PaO_2_/FiO_2_ (mm Hg) | 75 ± 12  (56-92) |  |  |  |  |  |  |  |  |  |  | 492 ± 85  (385-634) |  |  |  |  |  |  |  |
| PaCO_2_ (mm Hg) | 61 ± 10  (48-82) |  |  |  |  |  |  |  |  |  |  | 64 ± 23  (43-125) |  |  |  |  |  |  |  |

**Title of data**: Ventilatory settings and arterial blood gases in each experimental condition.

**Description of data**: Values are number of mean ± standard deviation (range).

ALI= acute lung injury onset; PEEP = positive en-expiratory pressure; RR = respiratory rate; V_T_ = tidal volume.
