# Supplementary material for: Reliability of the nitrogen washin-washout technique to assess end-expiratory lung volume at variable PEEP and tidal volumes
Source: Intensive Care Med Exp. 2014 Apr 9;2:10. doi: 10.1186/2197-425X-2-10 (PMC4512977; doi:10.1186/2197-425X-2-10)
Supplement: Supplementary file 4 — Additional file 4: Figure S2: Bias and limits of agreement between EELVCT and EELVWI-WO, using Bland and Altman representation in a subset of the data, acquired during the PEEP trial (with 10 min between measurements). Each symbol represents a concomitant measurement of EELVWI-WO and EELVCT. Horizontal continuous line and horizontal broken lines are the mean bias and 95% prediction interval limits of the bias between EELVWI-WO and EELVCT, respectively. EELVWI-WO, end-expiratory lung volume assessed with the nitrogen washin-washout technique; EELVCT, end-expiratory lung volume assessed by computed tomography; 95% p.i., 95% prediction interval of the bias between EELVWI-WO and EELVCT; V T, tidal volume. (DOCX 137 KB) [file 40635_2013_12_MOESM4_ESM.docx]

**Additional file 4**

**File name**: Additional file 4

**File format**: .pdf


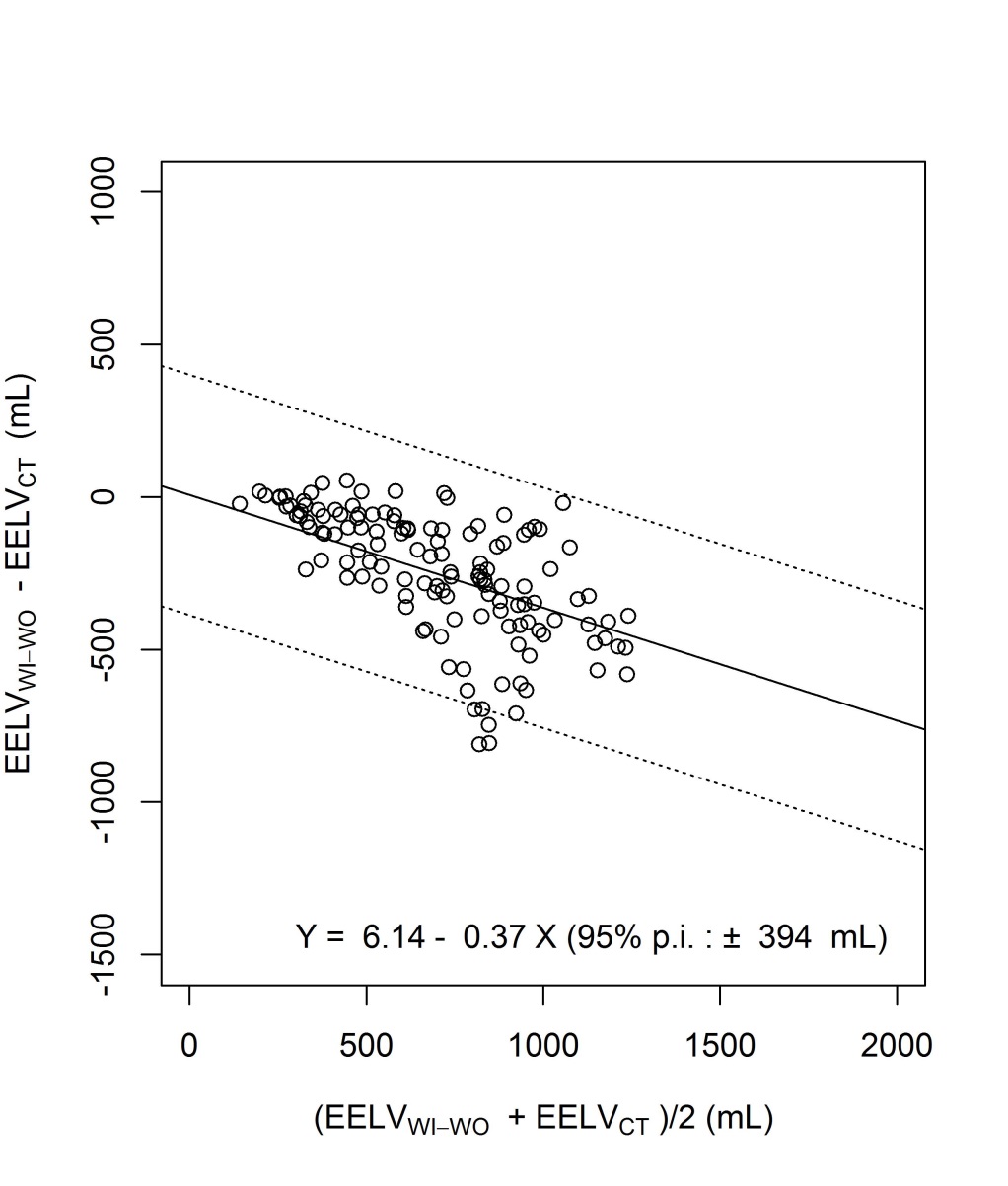


**Title of data**: Bias and limits of agreement between EELV_CT_ and EELV_WI-WO_, using Bland and Altman representation in a subset of the data, acquired during the PEEP trial (with 10-minutes between measurements).

**Description of data**: Each symbol represents a concomitant measurement of EELV_WI-WO_ and EELV_CT_. Horizontal continuous line and horizontal broken lines are the mean bias and 95% prediction interval limits of the bias between EELV_WI-WO_ and EELV_CT,_ respectively.

EELV_WI-WO_ = end-expiratory lung volume assessed with the nitrogen washout-washin technique; EELV_CT_ = end-expiratory lung volume assessed by computed tomography; 95% p.i. = 95% prediction interval of the bias between EELV_WI-WO_ and EELV_CT_; V_T_ = tidal volume.
